# Zufriedenheit mit der Weiterbildung an einem Level-I-Traumazentrum – Ergebnisse einer Umfrage und Entwicklung eines kompetenzbasierten Weiterbildungskonzeptes

**DOI:** 10.1007/s00104-024-02067-0

**Published:** 2024-03-18

**Authors:** J. Christoph Katthagen, Adrian Deichsel, Christian Schenk, Josef Stolberg-Stolberg, Johannes Glasbrenner, Michael J. Raschke

**Affiliations:** Klinik für Unfall‑, Hand- und Wiederherstellungschirurgie, Universitätsklinikum Münster, Albert-Schweitzer-Campus, Gebäude W1, 48149 Münster, Deutschland

**Keywords:** Weiterbildung, Mentoring, Kompetenz, Umfrage, Training, Residency, Further education, Mentoring, Competence, Survey, Training

## Abstract

**Hintergrund:**

Die strukturierte, kompetenzbasierte Weiterbildung ist einer der am häufigsten artikulierten Wünsche von AssistenzärztInnen.

**Methodik:**

Es erfolgte die Befragung von 19 AssistenzärztInnen hinsichtlich der Zufriedenheit mit der aktuellen Weiterbildung an einem Level-I-Traumazentrum, mittels eines Fragebogens mit 5 Fragen, welche auf einer 5‑Punkte-Likert-Skala beantwortet wurden. Im Folgenden erfolgte die Entwicklung eines überarbeiteten, kompetenzbasierten Weiterbildungskonzeptes.

**Resultate:**

Die Befragung spiegelte eine Unsicherheit wider, ob die aktuellen Weiterbildungsstrukturen den Anforderungen der Weiterbildungsordnung gerecht werden können. Das überarbeitete, kompetenzbasierte Weiterbildungskonzept besteht aus klinischem Mentoring, regelmäßigen theoretischen und praktischen Fortbildungen sowie regelmäßigen und strukturierten Mitarbeitergesprächen.

**Schlussfolgerung:**

Das vorgestellte Weiterbildungskonzept spiegelt den Versuch wider, eine zeitgemäße chirurgische Weiterbildung zu etablieren und sollte im Verlauf evaluiert werden.

## Abstract (en)

**Background:**

Structured competency-based training is one of the most frequently articulated wishes of residents.

**Methods:**

A survey of 19 residents was conducted regarding their satisfaction with the resident education at a level 1 trauma center. In this article the development of a revised competency-based education concept was carried out.

**Results:**

The survey reflected uncertainty as to whether the current structures could meet the requirements of the residency regulations. The improved competency-based education concept consists of clinical mentoring, competency-based catalogs of learning objectives, regular theoretical and practical workshops as well as regular and structured staff evaluations.

**Conclusion:**

The education concept presented reflects the attempt to establish a contemporary surgical training program which will be evaluated as it progresses.

## Hintergrund

Dem US-amerikanischen Chirurgen Dr. William S. Halsted, Gründer des ersten strukturierten Weiterbildungskurrikulums für AssistenzärztInnen in Amerika, wird das Zitat „See one, do one, teach one“ (kurz: SODOTO) zugeschrieben [15]. Glücklicherweise gehören solche rudimentären Weiterbildungskonzepte der Vergangenheit an und wurden im Laufe der Zeit signifikant überarbeitet [2, 26]. Operationsverfahren und einzelne Schritte werden heute durch strukturierte Kurssysteme und Trainingszentren vermittelt. Zunehmend haben auch Arbeitgeber Interesse an derartigen Formaten und unterstützen die Teilnahme der jüngeren AssistenzärztInnen, da gut ausgebildete, sichere MitarbeiterInnen eine deutliche Verbesserung der Behandlungsqualität, PatientInnensicherheit und Effizienz bedingt und zur Mitarbeiterzufriedenheit beiträgt. Obwohl die heutige chirurgische Weiterbildung, das wiederholte Trainieren operativer Eingriffe zur Erlangung von Expertise erfordert, ist die Weiterbildungssituation im deutschsprachigen Raum deutlich komplizierter. Ausufernde Bürokratisierung, Arbeitszeitbegrenzungen, Personalmangel sowie Zeit- und Qualitätsdruck an Krankenhäusern sind Gründe für verringerte Arbeits- und Weiterbildungszeit von AssistenzärztInnen an PatientInnen sowie im Operationssaal [10, 21]. Zusammengefasst ist, wie an vielen Stellen gefordert, die Weiterbildung junger AssistenzärztInnen eine der großen Herausforderungen der Orthopädie und Unfallchirurgie in der heutigen Zeit [12, 20, 32]. Zusätzlich ist die Finanzierung weiterbildender Maßnahmen bisher nicht klar im Gesundheitssystem geregelt.

**Figure 1:**
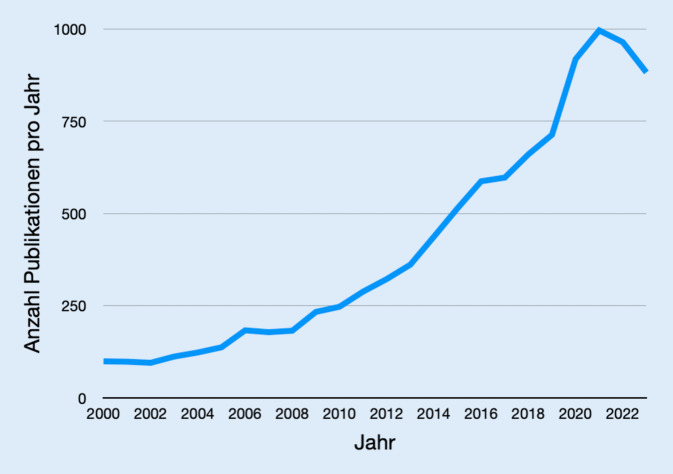

Umso wichtiger ist die Exploration und Etablierung moderner Weiterbildungskonzepte, welche inner- und außerhalb der Arbeitszeit eine adäquate und zeitgemäße Facharztweiterbildung ermöglichen. Das Interesse an zeitgemäßer Weiterbildung zeigt sich auch durch die gestiegene Anzahl an Publikationen in diesem Bereich. Durchsucht man PubMed nach den Suchbegriffen „(orthop* OR trauma) AND resident AND (train* OR simulat* OR educat*)“ zeigt sich im Vergleich zum Jahr 2000 eine Verzehnfachung der jährlichen Publikationen zu diesem Thema (Abb. 1).

Die Weiterbildung im Fach Orthopädie und Unfallchirurgie kann des Weiteren nicht mehr nur anhand reiner Zahlen gemessen werden, sondern am Erreichen von Kernkompetenzen, welche für die weitere ärztliche Laufbahn essenziell sind [9]. Dies spiegelt sich sowohl in der überarbeiteten Musterweiterbildungsordnung als auch in der Reform des Weiterbildungskatalogs für MedizinstudentInnen wider [9, 22, 31]. Die Kompetenzen gliedern sich dabei in kognitive und Methodenkompetenz (theoretische Kenntnisse) sowie Handlungskompetenz (praktische Erfahrungen/Fertigkeiten), welche notwendig sind, um chirurgische Problemstellungen erfolgreich und verantwortungsvoll zu lösen und somit für ein gutes Ergebnis für die PatientInnen zu sorgen [19].

Das Ziel dieses Artikels ist es, Lösungskonzepte für eine zeitgemäße, kompetenzbasierte, chirurgische Weiterbildung sowie die ergriffenen Maßnahmen darzustellen, ein strukturiertes Weiterbildungskurrikulum für AssistenzärztInnen an einem universitären und überregionalen Traumazentrum zu entwickeln.

## Methodik

### Evaluation der Weiterbildung

Die Weiterbildung an unserem überregionalen Traumzentrum wurde im Januar 2022 durch 19 WeiterbildungsassistentInnen evaluiert. Die erfassten Fragen waren: „Mit der aktuellen Struktur der Weiterbildung bin ich zufrieden“; „Im Rahmen meiner Weiterbildung wird auf den Erwerb von Kompetenzen geachtet“; „Aktuell kann ich gut einordnen, wie ich mit meinem Erwerb von Kenntnissen und Kompetenzen dastehe und ob ich die an mich gestellten Erwartungen erfülle“; „Aktuell habe ich gute Möglichkeiten, praktische ‚Alltagsfertigkeiten‘ unter Anleitung zu erwerben“; „Ich habe Sorgen, ob und wie ich den Inhalt der Weiterbildungsordnung erfüllen kann“. Die Fragen wurden auf einer 5‑Punkte-Likert-Skala (trifft zu, trifft eher zu, unentschieden, trifft eher nicht zu, trifft nicht zu) bewertet. Neben den Angaben bei den Fragen wurde das Alter, Geschlecht sowie der Weiterbildungsstand (1. bis 2. Weiterbildungsjahr [WJ], 3. bis 4. WJ, 5. bis 6. WJ) erfasst. Die Befragung erfolgte anonym.

Im Folgenden erfolgte die Entwicklung eines überarbeiteten Weiterbildungskonzeptes.

## Resultate

Die Verteilung der an der Umfrage teilnehmenden AssistentInnen war: 11 Personen WJ 1 bis 2 (58 %); 4 Personen WJ 3 bis 4 (21 %); 4 Personen WJ 5 bis 6 (21 %). Das Alter der teilnehmenden Personen war im Mittel 31 Jahre. Das Geschlechterverhältnis war ausgeglichen mit 10 männlichen sowie 9 weiblichen TeilnehmerInnen.

**Figure 2:**
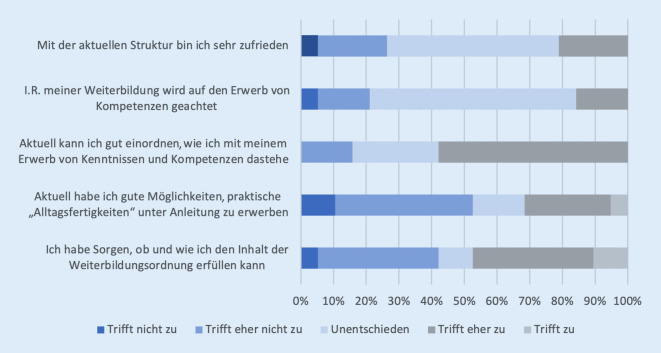

Insgesamt beantworteten 26 % der befragten AssistentInnen die Frage „I. R. meiner Weiterbildung wird auf der Erwerb von Kompetenzen geachtet“ mit „trifft nicht zu“ oder „trifft eher nicht zu“. Weitere 53 % waren unentschieden (Abb. 2). Die Frage „Aktuell habe ich gute Möglichkeiten, praktische ‚Alltagsfertigkeiten‘ unter Anleitung zu erwerben“ beantworteten 53 % der Befragten mit „trifft nicht zu“ oder „trifft eher nicht zu“ (11 % unentschieden). 48 % der Befragten gaben an, sich Sorgen zu machen, ob sie die Inhalte der Weiterbildungsordnung erfüllen können. Die Befragung der AssistentInnenschaft an unserer Klinik spiegelt den Wunsch nach einer Verbesserung der Weiterbildung dar.

### Entwicklung eines überarbeiteten Weiterbildungskonzeptes

#### Einführung kompetenzbasierter Lernzielkataloge und „entrustable professional activities“ je nach Weiterbildungsstand

Um eine stringente Weiterbildung zu ermöglich, müssen sowohl Weiterbildende als auch Weiterzubildende wissen, welche Anforderungen in welchem Weiterbildungsabschnitt an sie gestellt werden können sowie auf welche Fertigkeiten der Fokus der jeweiligen Weiterbildungsperiode gesetzt werden sollte. In diesem Themenkomplex spielen insbesondere die „entrustable profession activities“ (EPAs) eine wichtige Rolle. EPAs sind definiert als „Arbeitseinheit, die für den jeweiligen Arbeitsbereich typisch ist und die im Verlauf der Weiterbildung schrittweise dem Weiterzubildenden zur selbstständigen Durchführung anvertraut wird“ [4]. Dabei kann den Weiterzubildenden im Verlauf der Facharztweiterbildung schrittweise eigenständige Verantwortung bei der Bearbeitung ärztlicher Tätigkeiten (z. B. Schockraummanagement, operative Eingriffe) übertragen werden. Das Anvertrauen eigenständiger Durchführung erfolgt dabei durch die betreuenden OberärztInnen und kann gleichzeitig als Möglichkeit verwendet werden, den Weiterbildungsstand von AssistenzärztInnen zu evaluieren. Um die Erwartungen aller Beteiligten zu präzisieren, wurden Anforderungskataloge, gestaffelt nach den Weiterbildungsjahren 1 bis 2, 3 bis 4 sowie 5 bis 6, erstellt, welche sowohl operative als auch theoretische Fertigkeiten (z. B. körperliche Untersuchung, Schockraummanagement, Indikationsstellung) enthalten. Die individuellen Fertigkeiten werden dabei in drei Kategorien eingeteilt: A – Assistieren/Observieren; B – Durchführung unter Aufsicht; C – eigenständige Durchführung unter Aufsicht. Natürlich ist bei jedem operativen Eingriff von WeiterbildungsassistentInnen die Supervision und Präsenz eines Facharztes gewährleistet. Beispielhaft werden in den Jahren 1 bis 2 die Anlage externer Fixateure der Kategorie B zugeordnet, während in den Jahren 3 bis 4 Nagelosteosynthesen und in den Jahren 5 bis 6 komplexere Osteosynthesen mit in das Portfolio aufgenommen werden (Tab. 1). Die Anforderungskataloge sollen AssistentInnen und OberärztInnen zur Orientierung der individuellen Weiterbildung verwenden können. Individuelle Schwerpunkte und Expertisen einzelner Personen bleiben in diesem System natürlich unberücksichtigt und sollten individuell berücksichtigt werden. Unabhängig von den o. g. Kompetenzkatalogen, welche die Durchführung gesamter Prozeduren vorsehen, erfolgt die Durchführung von Teilschritten komplexer Prozeduren je nach Weiterbildungsstand [14]. Hierzu zählen beispielsweise operative Zugänge, Teilschritte von Repositionen/Osteosynthesen sowie bei Arthroskopien.

**Table 1:** 

	Assistieren/Observieren (A)	Assistierte Durchführung unter Aufsicht (B)	Eigenständige Durchführung unter Aufsicht (C)
WJ 1–2	Einfache Osteosynthesen	Anlage Fixateur externe/Thoraxdrainagen	Einfache Wundversorgungen
	Durchleuchtung HWS/Fuß	Schockraummanagement	Untersuchung großer Gelenke
WJ 3–4	Komplexe Frakturversorgung (Wirbelsäule/Gelenkfrakturen)	Einfache Osteosynthesen	Ausgedehnte Wundversorgungen
	Navigierte Eingriffe	Stellung von Operationsindikationen	Schockraummanagement
			Sehnennähte
			Beckenfixateur
WJ 5–6	–	Komplexe Frakturversorgung (Pilon/Tibiakopf)	Stellung einfacher Operationsindikationen
		Endoprothetik Hüfte/Knie	Kompartmentspaltungen

#### Mentorenprogramm

Losgelöst von der innerklinischen Weiterbildung wird jede/jeder Assistentin/Assistent, zusammen mit 2 weiteren AssistentInnen (aus unterschiedlichen Weiterbildungsständen) einem/einer MentorIn, welcher i. d. R. ein junger Oberarzt oder Facharzt ist, zugeordnet. Die Treffen der Mentorengruppe wurden intensiviert (einmal pro Quartal) mit dem Ziel, Probleme, Ziele und Meilensteine zu besprechen und Feedback zu geben. Es werden gegenseitig Tipps gegeben, welche Fallstricke es in den jeweiligen Karrierestufen gibt, welche Fortbildungen empfehlenswert sind und vieles mehr. Des Weiteren wird während des Mentorings ein besonderes Augenmerk auf die persönliche Entwicklung der AssistentInnen im Hinblick auf die Rolle des Arztes/der Ärztin gelegt (Entwicklung von Softskills). Hierbei können die anderen Gruppenmitglieder Feedback geben, wie die Person im Rahmen ihrer klinischen Tätigkeit auf andere wirkt und wie sich dies auf die PatientInnenversorgung auswirkt. Außerhalb der Treffen steht der/die MentorIn für Fragen zur Verfügung und hat im klinischen Alltag ein besonderes Auge auf seine Mentees.

#### Wöchentliche AssistentInnenfortbildung

Zusätzlich zur innerklinischen Weiterbildung erfolgte die Erarbeitung eines einjährigen Fortbildungskurrikulums, welches die Vermittlung der wichtigsten theoretischen Kenntnisse (Schulung kognitive und Methodenkompetenz) in Orthopädie und Unfallchirurgie zum Ziel hat. Das Fortbildungskurrikulum besteht aus insgesamt 52 Themen, welche nacheinander in wöchentlichem Abstand (Dauer ca. 1 Jahr) behandelt werden. Die Themenauswahl erfolgt dabei orientierend an den Inhalten der Weiterbildungsordnung für das Fach Orthopädie und Unfallchirurgie. Themenkomplexe des Fortbildungskurrikulums sind dabei unter anderem Transfusionsmedizin, Leichenschau, gelenkspezifische körperliche Untersuchung, operative Zugangswege sowie die Durchführung von Standardosteosynthesen. Die Vorbereitung der jeweiligen Fortbildung erfolgt im Team aus Assistenz‑, Fach- und Oberarzt, wobei der/die AssistentIn die ca. einstündige Präsentation für die KollegInnen übernimmt. Die Fortbildung findet in Randarbeitszeiten (16:00 bis 17:00) statt, um einer möglichst hohen Anzahl von Personen die Teilnahme zu ermöglichen. Zusätzlich findet im Rahmen der Morgenbesprechung ein wöchentlicher Journal Club statt, in welchem aktuelle orthopädische und unfallchirurgische Publikationen aufgearbeitet und mit dem gesamten Team diskutiert werden [29]. Hierbei wird neben dem Transfer aktueller Studienergebnisse ein besonderes Augenmerk auf die Methodik der diskutierten Studien gelegt, um eine Qualitätsbewertung zu schulen.

#### Praxiskurse an Trocken- oder Nasspräparaten/Simulatortrainings

**Figure 3:**
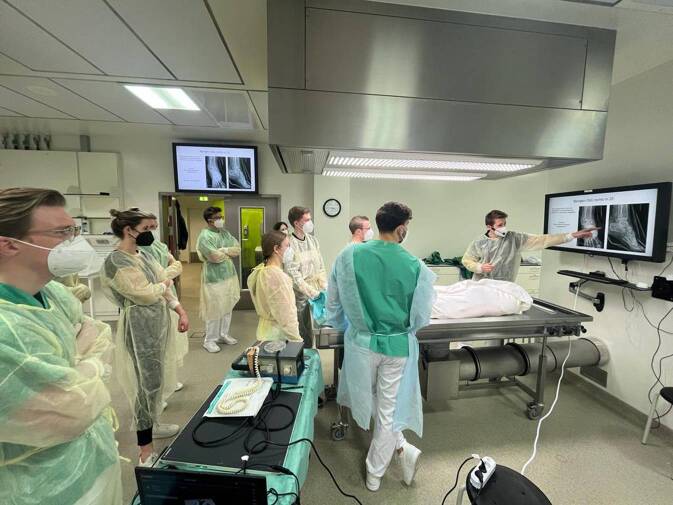

In regelmäßigen Abständen finden im Rahmen der Fortbildung praktische Workshops zu chirurgischen Basisfertigkeiten statt. Ziel der Praxiskurse ist die Schulung der Handlungskompetenz (Erwerb praktischer Fertigkeiten). Hierzu zählen Osteosynthese-Workshops an Sawbones, arthroskopische Dry-Labs, virtuelle Simulatortrainings sowie Trainings an Körperspenderpräparaten (Abb. 3). Die Teilnahme an den Basiskursen der Arbeitsgemeinschaft Osteosynthese (AO), Kursen zur Röntgendiagnostik sowie an ATLS-Kursen wird von der Klinik finanziell übernommen. Weitere Fortbildungen können auf Anfrage und plausibler Begründung unterstützt werden. Zusätzlich stehen an der Klinik Arthroskopie- sowie Osteosynthesesimulatoren (Dry-Labs) zur Verfügung. Diese können von den AssistenzärztInnen in eigener Regie zur Fortbildung verwendet werden. Dies erlaubt das Erlernen/Trainieren praktischer Fertigkeiten im „geschützten Raum“ und das Beheben von Fehlern ohne direkte Konsequenz für PatientInnen.

#### Strukturiertes Weiterbildungsgespräch

Die Evaluation des Erfolgs der individuellen Weiterbildung erfolgt im Rahmen des regelmäßigen Weiterbildungsgespräches, welches zweimal jährlich durch den Direktor der Klinik im Beisein eines leitenden/geschäftsführenden Oberarztes durchgeführt wird. Hier haben sowohl Weiterbildende als auch Weiterzubildende die Möglichkeit, zu artikulieren, welche Aspekte der Weiterbildung zum jetzigen Zeitpunkt gut laufen und welche noch Verbesserungen benötigen. Dabei bezieht sich das Feedback sowohl auf die Hardskills (operative Fähigkeiten) als auch auf die Softskills (Empathie, PatientInnenmanagement, Teamwork). Zusätzlich werden organisatorische Aspekte der Weiterbildung wie Rotationen besprochen. Die Ergebnisse des Weiterbildungsgesprächs werden schriftlich fixiert und stehen den Teilnehmenden zur Verfügung.

## Diskussion

Die strukturierte und optimierte Weiterbildung wurde von der AssistentInnenschaft vielfach als Notwendigkeit gefordert [12, 32]. In einer Umfrage des Jungen Forums Orthopädie und Unfallchirurgie (JFOU) mit 208 Medizinstudenten wurde eine strukturierte Weiterbildung als einer der wichtigsten Entscheidungsgründe für oder gegen eine Weiterbildungsstätte angegeben [13]. Die Anforderungen an die Weiterbildung in chirurgischen Fächern haben sich in den letzten Jahren deutlich verändert und weiterentwickelt. Die Weiterbildungskonzepte weiterbildender Einrichtungen sollten dies widerspiegeln und sich stetig den aktuellen Anforderungen anpassen [11]. Die Befragung eigener Mitarbeiter spiegelt jedoch die Unsicherheit wider, ob ein klassisches Weiterbildungskonzept den Anforderungen, welche an AssistenzärztInnen gestellt werden, gerecht werden kann. Dies entspricht dem Wunsch vieler junger ÄrztInnen in Deutschland nach verbesserter Struktur der Weiterbildung [20, 25]. Das dargestellte Konzept ist somit der Versuch, Weiterbildung zeitgemäßer zu gestalten, um den eigenen Mitarbeitenden eine umfassende theoretische und praktische Weiterbildung im Rahmen der Facharztweiterbildung zu ermöglichen.

Weiterbildung in Orthopädie und Unfallchirurgie bzw. chirurgischen Fächern im Generellen kann nicht ausschließlich an PatientInnen stattfinden. Extrakurrikuläre Veranstaltungen und Trainings in klinischen Randzeiten sollten zur Verfügung gestellt werden. Des Weiteren ist es wünschenswert, dass Skillslabs mit Trainingsmöglichkeiten für offene sowie endoskopische Eingriffe zur Verfügung stehen. Hier stellen insbesondere die modernen Simulationstechniken eine Chance dar.

### Erweiterung der Weiterbildung um digitale Simulation

Durch moderne Simulationstechniken haben sich in den vergangenen Jahren zahlreiche zusätzliche Modalitäten der chirurgischen Weiterbildung entwickelt, welche an dieser Stelle nicht unerwähnt bleiben sollten. Vorteile der chirurgischen Simulatoren sind die zeitliche Flexibilität sowie der geringe Materialaufwand (im Vergleich zu Trainings an Knochenmodellen oder Körperspenderpräparaten) während der Verwendung, sodass AssistentInnen ihr Training in ihrem eigenen Tempo, zu einem passenden Zeitpunkt in Eigenregie absolvieren können [7]. Jedoch steht diese Art der Weiterbildung an vielen Kliniken noch nicht zur Verfügung [5]. Denkbar wäre eine ggf. Ausweitung, die Optionen einer Einrichtung im Rahmen gemeinsamer Strukturen (z. B. Traumanetzwerke) zur Verfügung zu stellen.

Multiple Studien zeigen für endoskopische Simulatoren (Arthroskopie, Laparoskopie, Gynäkologie) positive Effekte auf die Performance von AssistentInnen in klinischen Szenarien [1, 24, 27, 28, 33]. Eine aktuelle Übersichtsarbeit, welche 44 Studien zur Wirksamkeit arthroskopischer Simulatoren für die Weiterbildung von AssistenzärztInnen evaluierte, stellte dar, dass 95 % der eingeschlossenen Studien einen positiven Einfluss von Simulatoren auf die arthroskopische Performance der AssistenzärztInnen zeigten [16]. Den positiven Aspekten stehen die in der Regel sehr hohe Anschaffungs- und Wartungskosten für digitale Simulationstrainer gegenüber.

Eine weitere neue Entwicklung stellen Simulatoren dar, welche sich Techniken der „Virtual Reality“ oder „Augmented Reality“ bedienen. Während der Verwendung wird in der Regel eine Brille getragen, welche die Umgebung vollständig oder teilweise durch digitale Simulationen ergänzt. Erste Studien, welche diese Techniken für die Weiterbildung in chirurgischen Fächern evaluierten, zeigen vielversprechende Ergebnisse. So zeigte eine randomisiert-kontrollierte Studie, welche Virtual-Reality-Training mit konventionellen Vorbereitungsmethoden für das Training der Implantation von Hüft-TEPs verglich, eine signifikante Überlegenheit der Virtual-Reality-Gruppe hinsichtlich der Präzision und Geschwindigkeit [17]. Ähnlich positive Effekte werden in weiteren Studien gesehen, jedoch ist die Datenlage bei dieser jungen Technik noch dünn [3, 18].

### „Don’t forget the classics“

Trotz des Enthusiasmus um die modernen Techniken sollten jedoch die klassischen Weiterbildungskonzepte nicht vernachlässigt werden. Die direkte Weiterbildung durch eine erfahrene Person an PatientInnen oder an lebensähnlichen Modellen kann nicht durch Simulationen ersetzt werden [6]. Zusätzlich ist das persönliche Mentoring von AssistenzärztInnen durch KollegInnen höherer Erfahrungsstufen unabdingbar für die persönliche und professionelle Weiterentwicklung [30]. Zuletzt sollte eine regelmäßige Evaluation des Weiterbildungsfortschrittes erfolgen, z. B. im Rahmen regelmäßiger Mitarbeitergespräche. Ergänzend können 360°-Feedbacks erfolgen, in denen eine Gruppe leitender ÄrztInnen einzelnen AssistenzärztInnen schriftliches Feedback geben [8, 23].

### Finanzierung der Weiterbildung

Derzeit wird der Aufwand, welcher von Weiterbildungsstätten betrieben wird, um eine Ausbildung der MitarbeiterInnen durchzuführen, nicht oder unzureichend vergütet. In Zeiten eines stetig steigenden ökonomischen Drucks wird Weiterbildung schnell zu einem Kostenfaktor. Es liegt an der Politik, zu gewährleisten, dass hochqualitative Weiterbildung in Zukunft explizit gefördert wird. Möglichkeiten wären z. B. die Einrichtung von Fortbildungsbudgets pro MitarbeiterIn, welche für weiterbildende Maßnahmen verwendet werden können.

### Limitationen

Bei der Interpretation der Ergebnisse der vorliegenden Studie sind mehrere Limitationen zu beachten. Zuerst handelt es sich bei den Ergebnissen der vorgestellten Umfrage lediglich um eine Stimmungsabfrage. Die Zufriedenheit mit spezifischen Inhalten der Weiterbildung wurde nicht abgefragt. Des Weiteren sollte die Befragung der AssistentInnen im Verlauf erneut durchgeführt werden, um einen möglichen Effekt des überarbeiteten Weiterbildungskonzeptes darzustellen. Die Überarbeitung des Weiterbildungskonzeptes erfolgte an mehreren Stellen (Einführung Lernzielkataloge, Intensivierung Mentoring, vermehrte Fortbildungen). Somit wird eine Veränderung in der Zufriedenheit mit der Weiterbildung nicht auf eine spezifische Intervention zurückzuführen sein.

## Schlussfolgerung

Das vorgestellte Weiterbildungskonzept spiegelt den Versuch wider, eine zeitgemäße chirurgische Weiterbildung zu etablieren und sollte im Verlauf evaluiert werden.
